# Maternal History and Uterine Artery Doppler in the Assessment of Risk for Development of Early- and Late-Onset Preeclampsia and Intrauterine Growth Restriction

**DOI:** 10.1155/2009/275613

**Published:** 2009-05-27

**Authors:** Elisa Llurba, Elena Carreras, Eduard Gratacós, Miquel Juan, Judith Astor, Angels Vives, Eduard Hermosilla, Ines Calero, Pilar Millán, Bárbara García-Valdecasas, Lluís Cabero

**Affiliations:** ^1^Department of Obstetrics, Fetal Medicine Unit, Vall d'Hebron Hospital, Universitat Autònoma de Barcelona, 08035-Barcelona, Spain; ^2^Department of Obstetrics, Son Llatze Hospital, Mallorca, Spain; ^3^Department of Obstetrics, Granoller Hospital, Barcelona, Spain; ^4^Department of Obstetrics, Terrassa Hospital, Barcelona, Spain; ^5^Department of Stadistics, Vall d'Hebron Hospital, Universitat Autònoma de Barcelona, 08035-Barcelona, Spain

## Abstract

*Objective*. To examine the value of one-step uterine artery Doppler at 20 weeks of gestation in the prediction pre-eclampsia (PE) and/or intrauterine growth restriction (IUGR). *Methods*. A prospective multicentre study that included all women with singleton pregnancies at 19–22 weeks of gestation (w). The mean pulsatility index (mPI) of both uterine arteries was calculated. Receiver-operating characteristics curves (ROC) were drawn to compare uterine artery Doppler and maternal risk factors for the prediction of early-onset PE and/or IUGR (before 32 w) and late-onset PE and/or IUGR. *Results*. 6,586 women were included in the study. Complete outcome data was recorded for 6,035 of these women (91.6%). PE developed in 75 (1.2%) and IUGR in 69 (1.1%) cases. Uterine Doppler mPI was 0.99 and the 90th centile was 1.40. For 10% false-positive rate, uterine Doppler mPI identified 70.6% of pregnancies that subsequently developed early-onset PE and 73.3% of pregnancies that developed early-onset IUGR. The test had a lower detection rate for the late-onset forms of the disease (23.5% for PE and 30% for IUGR). Maternal history has a low sensitivity in the detection of early-onset cases, although it is better at detecting late-onset PE. *Conclusion*. Uterine artery Doppler and maternal risk factors seem to select two different populations - early and late-onset PE which might suggest a different pathogenesis.

## 1. Introduction

Pre-eclampsia (PE) and intrauterine growth restriction (IUGR), which affect 4–10% of all pregnancies, are the leading cause of premature iatrogenic deliveries and maternal morbidity in developed countries [1]. One of the major goals of fetal-maternal medicine is to detect those cases at risk early enough in pregnancy to be able to tailor interventions to improve maternal and fetal outcomes.

Both PE and IUGR are associated with pathogenic evidence of placental underperfusion and ischemia [2, 3]. Blood flow through the uteroplacental circulation can be studied noninvasively using Doppler ultrasound. In pregnancies with PE or IUGR, impedance of flow in the uterine arteries increases before the clinical signs of the disease are seen [4, 5]. These findings have been supported by histologic studies that show that Doppler resistance index is inversely related to the percentage of vessels with trophoblastic invasion [6]. 

Implementation of uterine Doppler as a screening test has been limited due to the high variability in the sensitivity of PE, ranging from 30 to 80, together with a high false-positive rate [7]. The disparity of results when assessing efficacy of this screening test is due to differences in Doppler technique, the definition of abnormal flow velocity wave form, the population selected, or gestational age at the moment of screening [8]. In fact, the importance of distinguishing PE from transient hypertension in pregnancy or IUGR from constitutional small babies has only recently been considered [9]. Some studies used a two-stage program assessment [10–13], whereas nowadays one step evaluation at the moment of the anomaly scan (around 20 w in the majority of European countries) would be enough in clinical practice [14]. There are only two studies that evaluate uterine artery Doppler screening at 20 w of gestation [15, 16]; however, in both cases the number of patients was too small to draw meaningful conclusions. 

Therefore, the aim of our study was to examine the value of one-step uterine artery Doppler and maternal risk factors at 20 weeks of gestation in the prediction of early-onset and late-onset pre-eclampsia (PE) and/or intrauterine growth restriction (IUGR) in an unselected population. Moreover, we wanted to ascertain whether uterine artery Doppler is more effective in a high risk population.

## 2. Material and Methods

### 2.1. Study Population

This was a multicentric screening study involving Doppler ultrasound examination of the uterine arteries at 19–22 weeks of gestation in women with singleton pregnancies attending a routine second trimester anomaly scan. The study period was from June 2002 to May 2006.

The study was approved by the ethical committees of each hospital that took part. The participating hospitals were Hospital Son Llatzer de Mallorca, Hospital de Terrasa, Hospital de Granollers, and Hospital Vall d’Hebron de Barcelona (Spain). In these centers, all women who attended routine care were offered a transabdominal ultrasound examination at 19–22 weeks of gestation for measurement of fetal growth and examination for fetal abnormalities. All women with no major fetal abnormality were offered the option of uterine artery Doppler evaluation. Written consent was obtained in all cases. A first trimester scan was preformed in all patients, and CRL measurement was used to date the pregnancy. All sonographers were obstetricians specialized in fetal ultrasound. Quality control of screening, handling of data, and verification of adherence to protocols at the different centers were performed by the trial coordinators. Managing clinicians were blinded to the uterine Doppler measurements. As a result, there was no special follow-up for pregnant women with increased resistance in uterine arteries.

### 2.2. Uterine Artery Doppler Evaluation

Uterine artery Doppler velocimetry was evaluated at 19–22 weeks of gestation by abdominal ultrasound using 6–4 MHz probes Siemens Sonoline Antares (Siemens Medical, Erlangen, Germany); Acuson SP-10 (Acuson, Mountain View, Ca, USA); Aloka 5000 and Aloka 1700 (aloka, Tokyo, Japan); Toshiba SSH-140 (Toshiba, Tokyo, Japan). 

Flow velocity waveforms of the right and left uterine arteries were imaged with the patient semirecumbent, and the uterine artery was identified on a longitudinal scan, lateral to the uterus. In that position the scan showed the bifurcation of the common iliac artery. Recording was made at the point where the uterine artery and the external iliac artery appeared to have crossed each other, as detected by color flow Doppler. Pulsed wave Doppler was then used to obtain three consecutive waveforms. Following this, pulsatility index (PI) was measured, and the presence or absence of an early diastolic notch was noted. The process was then repeated for the contralateral uterine artery, and the mean PI (mPI) of the two vessels was calculated.

Maternal history risk factors were obtained prospectively. Patients were asked to complete a questionnaire on maternal age, race, height, weight, smoking status, obstetric history (previous PE, IUGR, abruption, or stillbirth), and medical history including chronic hypertension or diabetes. Maternal history risk factors were obtained from a questionnaire, although data were confirmed, when possible, by the examination of clinical history. Demographic characteristics and Doppler findings were recorded in a computer database at the time of Doppler studies in every participating center. Data on pregnancy outcomes were obtained from examination of each patient's clinical history and labor ward records.

For the purpose of this study both IUGR and PE were classified as early onset (gestational age under 32 weeks at delivery) or late onset (32 weeks or over). The classification of pre-eclampsia at or near to term has clinical importance since early-onset PE is commonly associated with IUGR, abnormal uterine and umbilical artery Doppler evaluation and adverse maternal and neonatal outcomes. In contrast to this, late-onset PE is mainly associated with a mild maternal disease and low rate of fetal involvement, and therefore perinatal outcome is usually favorable. Patients with severe early-onset PE have different risk factors compared to late-onset PE [17, 18]. Criteria for the definition of PE were those of the International Society for the Study of Hypertension in Pregnancy [19]. PE was diagnosed if a previously normotensive woman had her diastolic blood pressure above 90 mmHg measured twice (4 hours apart) and also had proteinuria of more than 300 mg in a 24-hour urine specimen or 2+ protein dipsticks twice (4 hours apart) after the 20th week of gestation. IUGR was diagnosed if the estimated fetal weight was below the 10th percentile for gestational age in our population, together with a Doppler PI in the umbilical artery above the 95th percentile, or if the estimated fetal weight was below the 3rd percentile irrespective of the umbilical artery Doppler [20]. 

### 2.3. Statistical Analysis

Mean PI was not normally distributed and therefore expressed as median ± interquartile range (IQR). Fisher's exact test was used to analyze maternal history variables, and independent *t*-test—Mann-Whitney *U* test—was used for continuous variables analysis where appropiate.

The sensitivity (S), specificity (E), positive predictive value (PPV), negative predictive value (NPV), and likelihood ratio (LR) for a cut-off mean PI of 1.66 (95th centile) and for a mean PI of 1.40 (90th centile) and presence of bilateral notches in the prediction of preeclampsia and/or IUGR were calculated. Differences were considered significant when *P* < .05. 

Logistic regression was used to obtain the odds ratio (OR) and 95% CI for PE in relation to maternal history variables. A multivariate analysis to draw the receiver-operating characteristics curves (ROC) was performed using maternal history variables found to be independent in a univariate analysis. 

The sensitivity and specificity for different cut-offs in marker levels were calculated, and ROC were drawn to compare the performances of uterine Doppler and maternal risk factor tests. ROC analysis was also used to compare uterine artery Doppler in low- and high-risk populations.

Data was analyzed using SPSS 13.0 (SPSS, Chicago, Ill, USA) and STATA/SE 8.2 statistical packages.

## 3. Results

Doppler examination of the uterine arteries was attempted in 6586 consecutive singleton pregnancies. Satisfactory waveforms were obtained from both vessels in 6535 cases (99%). Complete outcome data was recorded in 6035 cases (91.6%), which formed the population for our study. No differences were found in the demographic and screening characteristics of the 6035 pregnancies with follow-up and those lost for follow-up, except for their smoking status, that was significantly higher in the group with complete follow-up (data not shown). In Table 1 we presented the maternal risk factors for the development of PE. There was an increased risk for PE in nulliparus, in noncaucasian women, in women with a body mass index (BMI) >30 and in those with a history of hypertension, previous PE, stillbirth, or abruption. The risk of PE decreased in cigarette smokers.

**Figure 1:**
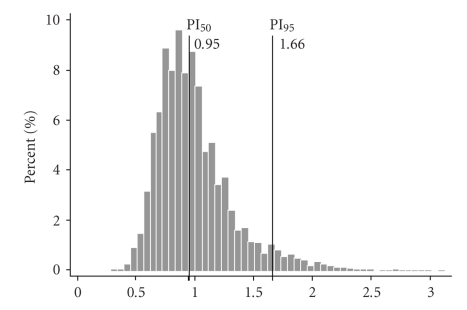
Frequency distribution of mean Pulsatility Index (mPI) uterine artery Doppler in the study population.

Uterine artery mPI was not normally distributed but was found to be skewed to the right with a mean value of 0.99 and the 95th percentile of 1.66 (Figure 1). In the group of women who subsequently developed PE/IUGR, mPI was higher than in those who did not develop any complication (median 1.36 (0.70) versus 0.93 (0.30), *P* < .001). The highest values of mPI were among patients that developed early-onset disease (median 1.68 (0.40) versus 1.31 (0.43), *P* < .01). 

Overall, PE occurred in 75 cases (1.2%), early-onset PE in 20 cases (0.3%), IUGR in 69 cases (1.1%); early-onset IUGR in 33 cases (0.5%) and early-onset PE with IUGR in 38 cases (0.6%). Screening characteristics for early-onset PE/IUGR between uterine artery mPI > 1.66 (95th percentile) and/or bilateral notches are set out in Table 2. Uterine artery mPI > 1.40 (90th percentile) was able to detect 73.7% of early-onset PE/IUGR with the same false-positive rate (10%) as mPI > 95th plus bilateral notches, as shown in Table 3. 

**Figure 2:**
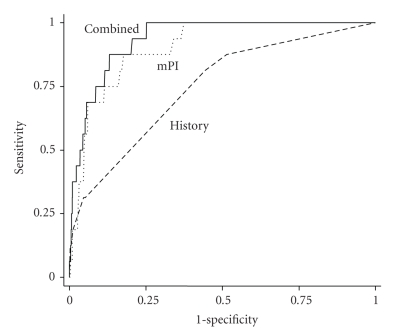
Receiver-operating characteristic curves showing the sensitivities for given screen-positive rates of uterine Doppler mean PI assessment (mPI), maternal history characteristics (History) and the combination of both methods (Combined) in the detection of early-onset PE.

**Figure 3:**
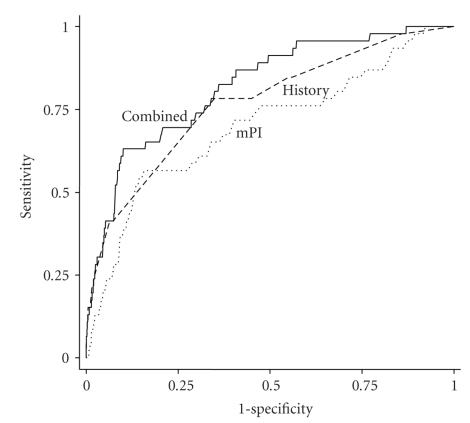
Receiver-operating characteristic curves showing the higher sensitivities for given screen-positive rates of uterine Doppler mean PI assessment (mPI), maternal history characteristics (History) and the combination of both methods (Combined) in the detection of late-onset PE.

In order to perform a combined assessment, uterine artery mPI was compared with the maternal history variables in women who subsequently developed PE and those who did not. Moreover, we were able to differentiate early- and late-onset PE by drawing ROC curves to compare the two methods of screening for early-onset PE (Figure 2) and late-onset PE (Figure 3). By comparing the areas under the curves, it was shown that uterine Doppler mPI (AUC = 0.90, 95% CI (0.85–0.96) *P* < .001) seemed to perform better than maternal history alone (AUC = 0.75, 95% CI (0.63–0.87) *P* = .001) in detection of early-onset PE, although there was no statistical difference when we compared both AUCs (*P* = .076). The performance of uterine Doppler mPI assessment of risk (AUC = 0.70, 95% CI (0.61–0.78) *P* < .001) was not statistically different from maternal history (AUC = 0.77, 95% CI (0.69–0.84, *P* < .001) for late-onset PE (*P* = .254). The combination of the two methods did little to increase the sensitivity of either early- or late-onset PE. Uterine artery Doppler performance for risk assessment of early-onset PE in the low-risk population (AUC = 0.91, 95% CI (0.850–0.972) *P* < .002) and in the high-risk population (AUC = 0.87, 95% CI (0.718–1.00) *P* = .062) showed no differences (*P* = .53) in detection of early-onset PE.

## 4. Discussion

The results of this study demonstrate that uterine artery Doppler is an effective screening program for the prediction of PE and IUGR in an unselected population with classically low incidence of PE. Our findings have shown that measurement of the uterine artery mPI at 20 weeks gestation can identify 70.6% of pregnancies that will subsequently develop early-onset PE and 73.3% of pregnancies that develop early-onset IUGR, with a 10% false-positive rate.

In our screening program, the gestational age was moved to 19–22 weeks, which is when the routine abnormality scan is normally performed in most countries. Although a two-stage screening program has been recommended due to a higher false-positive rate around 20 weeks of gestation [21], we have found that, for the same false-positive rate, a one-stage screening test at 20 weeks gestation has a similar detection rate for PE or IUGR to studies performed at 22–24 weeks gestation [9].

We have also determined that with the same false-positive rate (10%), uterine Doppler mPI gave similar results to bilateral notches and mPI, so the addition of bilateral notches did nothing to improve screening characteristics. Moreover, the use of the PI has removed any subjectivity associated with the definition and interpretation of the presence of notches in the waveform [7].

While there is no effective therapeutic measure for the prevention of PE and/or IUGR, NICE guideline recommendations point out that a woman's degree of risk for PE should be evaluated so that an appropriate plan for subsequent scheduling of antenatal appointments [22] and ultrasound growth scans can be formulated. There are no direct iatrogenic procedures that could affect mother or fetus after being classified as high risk for these conditions, so it would be reasonable to maximize the detection rate even at the cost of a slightly higher screen-positive rate. Nonetheless, such a classification may cause anxiety even though increased surveillance can improve maternal and neonatal outcome. We have found that uterine Doppler mPI had a good sensitivity even in the low-risk population, precisely, the group of patients that benefit most from a program of specific follow-up.

We have considered early-onset PE and IUGR as different outcomes from those conditions that were diagnosed near to term. Previous studies have established that uterine Doppler is much better at identifying the more severe early-onset cases [14, 23–26]. It has also been demonstrated that uterine Doppler screening is better in predicting severe early-onset disease or PE associated with IUGR, with sensitivities of 80 to 90% [9]. To add to this, we have shown that uterine Doppler mPI performed better than maternal history in the detection of early-onset PE, whereas maternal history alone is similar to uterine Doppler mPI in the detection of late-onset PE. The combination of both methods did not significantly improve the sensitivity in early- or late-onset disease. We are aware of the limitations of maternal historical data as this piece of information relies on medical records and maternal self-reporting.

The classification of PE at or near term has clinical importance since early-onset PE is commonly associated with IUGR, abnormal uterine Doppler, and adverse maternal and neonatal outcomes [17, 27–29]. In contrast, late-onset PE is mainly associated with a milder form of maternal illness and low rate of fetal involvement, so perinatal outcome is usually favorable [17, 28]. Patients with severe early-onset PE have different risk factors compared to late-onset PE [29, 30]. Moreover, we have recently demonstrated that patients with early-onset PE (<32 weeks) had abnormal uterine Doppler mPI, whereas, in late-onset PE (>32 weeks), only a proportion of these cases presented abnormal uterine Doppler assessment, suggesting that there is a subgroup of late-onset cases with minimal placental involvement [28]. 

In conclusion, our results now show that a single 20 weeks uterine Doppler assessment was feasible and useful and is able to detect pregnant women with a high risk for early-onset adverse outcomes that relate to impaired trophoblast invasion in the low- and high-risk populations. In women with late-onset PE, maternal history and uterine Doppler are less effective in identifying those women at risk, signifying that late-onset PE could result from heterogeneous causes in the context of absent or minimal placental impairment. These findings require a shift away from the general concept of PE as a single entity toward defining different types of PE classified according to cause. The application of this screening test in routine clinical practice needs future studies to demonstrate that uterine artery Doppler is useful to identify the population with a higher risk to pregnancy complications for whom intensive surveillance could improve maternal and fetal health.

## Figures and Tables

**Table 1:** ORs for risk factors for the development of PE in the maternal history.

Characteristic		*N* (%)	OR	CI 95%	*P*
Age	>34	17 (1.5)	1.33	(0.77–2.30)	.322
Nulliparus		46 (1.9)	2.52	(1.53–4.14)	<.001
Race other than white		13 (1.6)	1.32	(0.72–2.41)	.386
BMI	≥30	17 (2.4)	2.39	(1.37–4.15)	.002
Smoker	Yes	7 (0.5)	0.33	(0.15–0.72)	.001
Chronic hypertension	Yes	10 (10.9)	9.22	(4.60–18.5)	<.001
Diabetes I/II	Yes	4 (4.5)	4.5	(1.38–10.8)	<.031
Previous PE	Yes	9 (11.1)	11.22	(5.38–23.4)	<.001
Previous IUGR	Yes	3 (4.5)	3.91	(1.2–12.7)	.024
Previous abruption	Yes	1 (4.2)	3.5	(1.4 + 10.9)	.306
Previous stillbirth	Yes	4 (3.6)	3.06	(1.1–8.55)	.032

**Table 2:** Screening characteristics for mean PI > 1.66 and/or bilateral Notches at 20 w (screen positive rate: 9.2%).

	S (%)	E (%)	PPV (%)	NPV (%)	LR+	LR−
PE	48.0	91.3	6.5	99.3	5.5	0.18
PE < 32 w	75.0	91.0	2.7	99.9	8.3	0.12
PE ≥ 32 w	38.9	91.0	3.8	99.4	4.3	0.23
IUGR	52.2	91.3	6.5	99.4	6.0	0.17
IUGR < 32 w	72.7	91.1	4.3	99.8	8.2	0.12
IUGR ≥ 32 w	33.3	90.9	2.2	99.6	3.7	0.27
PE and/or IUGR < 32	73.7	91.2	5.0	99.8	8.4	0.12
PE and IUGR	65.2	91.0	2.7	99.9	7.2	0.14
PE and IUGR < 32 s	73.3	90.9	2.0	99.9	8.1	0.12
PE and IUGR ≥ 32 s	50.0	90.8	0.7	99.9	5.5	0.18

**Table 3:** Screening characteristics for mean PI > 1.40 irrespective of bilateral Notches at 20 w (screen positive rate: 10%).

	S (%)	E (%)	PPV (%)	NPV (%)	LR+	LR−
PE	46.0	90.0	5.2	99.3	4.7	0.21
PE < 32 w	70.6	90.0	2.0	99.9	7.4	0.14
PE ≥ 32 w	39.2	90.0	3.3	99.4	3.9	0.20
IUGR	57.1	90.3	5.9	99.5	6.0	0.17
IUGR < 32 w	73.3	90.1	3.6	99.9	7.4	0.13
IUGR ≥ 32 w	42.4	90.0	3.3	99.4	3.9	0.25
PE and/or IUGR < 32	71.4	90.0	4.1	99.8	7.3	0.14
PE and IUGR	68.4	90.0	2.1	99.9	6.8	0.15
PE and IUGR < 32 s	75.0	89.9	1.5	99.9	7.5	0.13
PE and IUGR ≥ 32 s	57.1	89.9	0.7	99.9	5.6	0.18
